# Clinical Features and Outcomes of Pediatric and Adult Patients Hospitalized for Coronavirus Disease 2019: A Comparison Across Age Strata

**DOI:** 10.1093/ofid/ofae443

**Published:** 2024-08-10

**Authors:** Grace X Li, Komal Gopchandani, Noah Brazer, Ashley Tippett, Chris Choi, Hui-Mien Hsiao, Miriam Oseguera, Abiodun Foresythe, Sanchita Bhattacharya, Venice Servellita, Alicia Sotomayor Gonzalez, Jennifer K Spinler, Mark D Gonzalez, Dalia Gulick, Colleen Kraft, Vyjayanti Kasinathan, Yun F (Wayne) Wang, Jennifer Dien Bard, Pei Ying Chen, Jessica Flores-Vazquez, Audrey R Odom John, Paul J Planet, Sridevi Devaraj, Ananth V Annapragada, Ruth Ann Luna, Charles Y Chiu, Christina A Rostad

**Affiliations:** Department of Pediatrics, Emory University School of Medicine, Atlanta, GA, USA; Department of Pediatrics, Emory University School of Medicine, Atlanta, GA, USA; Department of Laboratory Medicine, Division of Infectious Diseases, University of California at San Francisco, San Francisco, CA, USA; Department of Medicine, Division of Infectious Diseases, University of California at San Francisco, San Francisco, CA, USA; Department of Pediatrics, Emory University School of Medicine, Atlanta, GA, USA; Department of Pediatrics, Emory University School of Medicine, Atlanta, GA, USA; Department of Pediatrics, Emory University School of Medicine, Atlanta, GA, USA; Department of Laboratory Medicine, Division of Infectious Diseases, University of California at San Francisco, San Francisco, CA, USA; Department of Medicine, Division of Infectious Diseases, University of California at San Francisco, San Francisco, CA, USA; Department of Laboratory Medicine, Division of Infectious Diseases, University of California at San Francisco, San Francisco, CA, USA; Department of Medicine, Division of Infectious Diseases, University of California at San Francisco, San Francisco, CA, USA; Bakar Computational Health Sciences Institute, University of California, San Francisco, CA, USA; Department of Laboratory Medicine, Division of Infectious Diseases, University of California at San Francisco, San Francisco, CA, USA; Department of Medicine, Division of Infectious Diseases, University of California at San Francisco, San Francisco, CA, USA; Department of Laboratory Medicine, Division of Infectious Diseases, University of California at San Francisco, San Francisco, CA, USA; Department of Medicine, Division of Infectious Diseases, University of California at San Francisco, San Francisco, CA, USA; Department of Pathology, Texas Children’s Hospital, Houston, TX, USA; Department of Pathology & Immunology, Baylor College of Medicine, Houston, TX, USA; Department of Pathology, Children’s Healthcare of Atlanta, Atlanta, GA, USA; Georgia Clinical & Translational Science Alliance, Emory University School of Medicine, Atlanta, GA, USA; Department of Medicine, Division of Infectious Diseases, Emory University School of Medicine, Atlanta, GA, USA; Department of Pathology and Laboratory Medicine, Emory University School of Medicine, Atlanta, GA, USA; Department of Pathology and Laboratory Medicine, Emory University School of Medicine, Atlanta, GA, USA; Department of Pathology and Laboratory Medicine, Emory University School of Medicine, Atlanta, GA, USA; Clinical Microbiology Laboratory, Grady Memorial Health Center, Atlanta, GA, USA; Department of Pathology and Laboratory Medicine, Children's Hospital Los Angeles, Los Angeles, CA, USA; Keck School of Medicine, University of Southern California, Los Angeles, CA, USA; Department of Pathology and Laboratory Medicine, Children's Hospital Los Angeles, Los Angeles, CA, USA; Keck School of Medicine, University of Southern California, Los Angeles, CA, USA; Department of Pathology and Laboratory Medicine, Children's Hospital Los Angeles, Los Angeles, CA, USA; Keck School of Medicine, University of Southern California, Los Angeles, CA, USA; Department of Pediatrics, Children’s Hospital of Philadelphia, Philadelphia, PA, USA; Perelman School of Medicine, University of Pennsylvania, Philadelphia, PA, USA; Department of Pediatrics, Children’s Hospital of Philadelphia, Philadelphia, PA, USA; Perelman School of Medicine, University of Pennsylvania, Philadelphia, PA, USA; Department of Pathology, Texas Children’s Hospital, Houston, TX, USA; Department of Pathology & Immunology, Baylor College of Medicine, Houston, TX, USA; Department of Radiology, Baylor College of Medicine, Houston, TX, USA; Department of Radiology, Texas Children's Hospital, Houston, TX, USA; Department of Pathology, Texas Children’s Hospital, Houston, TX, USA; Department of Pathology & Immunology, Baylor College of Medicine, Houston, TX, USA; Department of Laboratory Medicine, Division of Infectious Diseases, University of California at San Francisco, San Francisco, CA, USA; Department of Medicine, Division of Infectious Diseases, University of California at San Francisco, San Francisco, CA, USA; Department of Pediatrics, Emory University School of Medicine, Atlanta, GA, USA; Department of Pediatrics, Children’s Healthcare of Atlanta, Atlanta, GA, USA

**Keywords:** adults, children, coronavirus, coronavirus disease 2019, SARS-CoV-2

## Abstract

**Background:**

Coronavirus disease 2019 (COVID-19) continues to cause hospitalizations and severe disease in children and adults.

**Methods:**

This study compared the risk factors, symptoms, and outcomes of children and adults hospitalized for COVID-19 from March 2020 to May 2023 across age strata at 5 US sites participating in the Predicting Viral-Associated Inflammatory Disease Severity in Children with Laboratory Diagnostics and Artificial Intelligence consortium. Eligible patients had an upper respiratory swab that tested positive for severe acute respiratory syndrome coronavirus 2 by nucleic acid amplification. Adjusted odds ratios (aOR) of clinical outcomes were determined for children versus adults, for pediatric age strata compared to adolescents (12–17 years), and for adult age strata compared to young adults (22–49 years).

**Results:**

Of 9101 patients in the Predicting Viral-Associated Inflammatory Disease Severity in Children with Laboratory Diagnostics and Artificial Intelligence cohort, 1560 were hospitalized for COVID-19 as the primary reason. Compared to adults (22–105 years, n = 675), children (0–21 years, n = 885) were less commonly vaccinated (14.3% vs 34.5%), more commonly infected with the Omicron variant (49.5% vs 26.1%) and had fewer comorbidities (*P* < .001 for most comparisons), except for lung disease (*P* = .24). After adjusting for confounding variables, children had significantly lower odds of receiving supplemental oxygen (aOR, 0.57; 95% confidence interval, .35–.92) and death (aOR, 0.011; 95% confidence interval, <.01–.58) compa­­red to adults. Among pediatric age strata, adolescents 12–17 years had the highest odds of receiving supplemental oxygen, high-flow oxygen, and ICU admission. Among adults, those 50–64 years had the highest odds of mechanical ventilation and ICU admission.

**Conclusions:**

Clinical outcomes of COVID-19 differed across pediatric and adult age strata. Adolescents experienced the most severe disease among children, whereas adults 50–64 years experienced the most severe disease among adults.

As of 26 February 2024, coronavirus disease 2019 (COVID-19) had caused >6.8 million hospitalizations and 1.1 million deaths in the United States alone [1]. Since 11 December 2020, the Pfizer-BioNTech COVID-19 vaccine has been available for individuals 16 years of age and older, and since 18 June 2022, the Centers for Disease Control and Prevention has recommended the COVID-19 vaccine for children as young as 6 months. Studies of real-world vaccine effectiveness have demonstrated that COVID-19 vaccines significantly reduce the risk of hospitalizations, intensive care unit (ICU) admissions, need for mechanical ventilation, and COVID-19–related mortality in older adults and children [2, 3].

However, both older adults and children remain at risk for severe COVID-19, with adults ≥65 years of age comprising 63% of COVID-19–associated hospitalizations in the US COVID-NET surveillance from January to August 2023 [4]. Although children with COVID-19 are hospitalized less frequently, those who require hospitalization often experience severe outcomes. Additionally, children with COVID-19 are at risk of developing the hyperinflammatory syndrome, Multisystem Inflammatory Syndrome in Children (MIS-C) [5].

Most published studies have focused on the characteristics of severe COVID-19 in either children or adults. Comorbidities such as chronic lung disease, prematurity, neurological disease, and cardiovascular disease have been associated with severe COVID-19 [6, 7] in younger children, whereas obesity and diabetes have been found to increase risk of hospitalization and COVID-19 severity in older children [7, 8]. In adults, obesity, hypertension, diabetes, and respiratory disease are associated with hospitalization [9, 10], whereas older age, male gender, and underlying comorbidities are associated with higher in-hospital mortality [11, 12]. Fever is the most commonly reported symptom among hospitalized children and adults; gastrointestinal and respiratory symptoms are also common [10, 13].

Data on the comparison of clinical characteristics, risk factors, and outcomes between children and adults hospitalized with COVID-19 are currently limited. The primary objective of this analysis was to compare clinical features and outcomes between children and adults hospitalized with COVID-19 using data collected through a multi-institutional consortium. This study further aimed to compare differential COVID-19 outcomes across different age strata in hospitalized pediatric and adult patients.

## METHODS

### Subject Population and Design

This was a retrospective analysis of a cohort of children and adults who tested positive for severe acute respiratory syndrome coronavirus 2 (SARS-CoV-2) by clinical upper respiratory specimen nucleic acid amplification testing from March 2020 to May 2023 at 5 participating sites within the National Institutes of Health consortium for Predicting Viral-Associated Inflammatory Disease Severity in Children with Laboratory Diagnostics and Artificial Intelligence (PreVAIL). Patients were eligible for inclusion in this PreVAIL cohort if they had a positive SARS-CoV-2 test by clinical upper respiratory nucleic acid amplification testing and residual specimen available for sequencing, regardless of their clinical symptoms or site of testing (eg, inpatient, outpatient, emergency department). The study sites included Children's Healthcare of Atlanta and Emory University–associated hospitals, Children's Hospital of Los Angeles (CHLA), Children's Hospital of Philadelphia, Texas Children's Hospital, and the University of California, San Francisco (UCSF). Pediatric patients were included at each of the participating sites, whereas adult patients were included at affiliated hospitals of Emory University and UCSF, and 2 adults were included at CHLA. In PreVAIL, the residual clinical upper respiratory samples were subjected to viral whole-genome sequencing to determine the infecting SARS-CoV-2 variant and clinical data were abstracted from the medical record and stored in a secure REDCap database. Detailed information about sample collection is included in the Supplementary Methods. For this manuscript, we performed a secondary analysis of those patients included in the PreVAIL study who were hospitalized for COVID-19 as the primary reason, regardless of whether the infecting SARS-CoV-2 variant was successfully identified by viral whole-genome sequencing.

### Patient Consent Statement

The procedures followed were in accordance with the ethical standards of the Helsinki Declaration (1964, amended most recently in 2008) of the World Medical Association. The local institutional review boards at each participating institution (CHLA, Children's Healthcare of Atlanta, Children's Hospital of Philadelphia, Emory, Texas Children's Hospital, UCSF) approved this study as “no subject contact” with waiver of informed consent for collection of residual swab samples and relevant metadata.

### Clinical Metadata

For all patients in the PreVAIL study, the study team abstracted clinical metadata, including demographic, clinical, and laboratory data from the electronic medical records by manual chart review. Data were stored in a secure, shared REDCap database. Demographic variables of interest in our analysis population included age, sex, race, and Latinx ethnicity. The underlying comorbidities considered included autoimmune disease, active malignancy, other immunocompromising condition (for example, human immunodeficiency virus/acquired immunodeficiency syndrome [HIV/AIDS], bone marrow or solid organ transplant, etc.), hypertension, obesity, diabetes, cardiac disease, and lung disease (for example, asthma, obstructive sleep apnea, etc.). Clinical variables collected at presentation included COVID-19 symptoms such as fever, chills, headache, fatigue, cough, shortness of breath/dyspnea, nasal congestion/rhinorrhea, sore throat, myalgias, nausea/vomiting, diarrhea, conjunctivitis, and anosmia/dysgeusia. We collected information on co-infections and categorized them as follows: bacteremia/sepsis, bacterial pneumonia, respiratory viral, gastrointestinal, skin/soft tissue, urinary tract infection, multiple, other respiratory, other viral, and other based on clinical diagnoses in the medical record. Microbial etiologies were recorded when present. We did not differentiate between co-infections that were preexisting before hospitalization versus those that were hospital-acquired. Thus, these were not regarded as outcomes, but were considered independent variables potentially modifying patient outcomes. We also collected vaccination status (defined as number of COVID-19 vaccines received before diagnosis), vaccine brand, and bivalent booster information. Because the COVID-19 vaccine was approved for children during the duration of the study period and there was not equal opportunity for all children to receive a booster or vaccine, we defined vaccination status as a binary variable based on whether the patient had received any COVID-19 vaccine in the final analysis.

The SARS-CoV-2 variants identified via whole-genome sequencing were classified as Omicron (BA.1 and its sublineages BA.2, BA.2.12.1, BA.3, BA.4, BA.5, XE, BQ.1, BQ.1.1, BF.7., XBB. and XBD) and non-Omicron (all other sequences and indeterminate results). Severity outcomes considered in our analysis included duration of hospitalization, utilization of supplemental oxygen (any), high-flow supplemental oxygen (a subset of those who received any supplemental oxygen), admission to ICU, utilization of mechanical ventilation, coinciding diagnosis of MIS-C or multisystem inflammatory syndrome in adults, and death.

### Statistical Analysis

Baseline characteristics of our analysis population were summarized using descriptive statistics. The 5 pediatric age strata included infants <1 year of age, children 1–4 years, 5–11 years, 12–17 years, and 18–21 years. The 4 adult age strata included adults 22–49 years, 50–64 years, 65–74 years, and ≥75 years. Study groups and age strata were compared using chi-square tests or 1-way analysis of variance. Sensitivity analyses were performed by classifying missing symptom data as either “unknown” and excluded or “no” and included (Supplementary Table 1). Subgroup analysis of symptoms in infants <3 months of age, who are often hospitalized per febrile infant pathways, was also performed (Supplementary Table 2). By univariate analysis, odds ratios for clinical outcomes of interest were determined for children versus adults (reference), and for each pediatric age stratum compared to children 12–17 years (reference), and for each adult age stratum compared to adults 22–49 years (reference). Multivariate logistic regression analysis was performed to determine odds ratios after adjusting for age, sex, race, Latinx ethnicity, vaccination status (yes/no), Omicron infection (yes/no), co-infection (yes/no), and study site. All analyses were performed using SAS v9.4 software.

## RESULTS

### Study Cohort

Of 9101 patients in the PreVAIL cohort who tested positive for SARS-CoV-2 and had residual samples available for viral sequencing, 2229 (24.5%) were hospitalized and 1560 (17.1%) were hospitalized for COVID-19 as the primary reason. Of the 1560 patients who were hospitalized for COVID-19 as a primary reason across the 5 institutions (the analysis population), 885 (56.7%) were children and 675 (43.3%) were adults (Supplementary Figures 1 and 2).

### Comparisons of Children and Adults Hospitalized for COVID-19

The median age of children in the analysis population was 5.8 years (interquartile range, 0.7–14.2), and the majority were Latinx ethnicity (440, 52.9%). Compared to adults, children were less commonly vaccinated (14.3% vs 34.5%), were more commonly infected with the Omicron variant (49.5% vs 26.1%), and had fewer comorbidities (*P* < .001 for nearly all comparisons), except for lung disease (24.4% vs 27.0%, *P* = .237) (Table 1). Regarding symptoms, children more commonly had fever, nasal congestion/rhinorrhea, conjunctivitis, and nausea/vomiting but less commonly had chills, headache, fatigue, cough, shortness of breath, and anosmia/dysgeusia (*P* < .05 for all comparisons). In a sensitivity analysis in which missing data were classified as lack of symptoms (“no” instead of “unknown,” Supplementary Table 1), statistically significant differences between children and adults were similarly observed. Of the 885 children in the analysis population, 163 (18.4%) were infants aged <3 months. The majority of these infants had respiratory symptoms in addition to fever, and their symptom profiles were similar to those of older children (Supplementary Table 2). Overall, children less commonly had co-infections (*P* = .001), and the most common pediatric co-infections were other respiratory viruses (40.7%; Supplementary Table 3). Although children less commonly received supplemental oxygen (*P* < .001), high-flow supplemental oxygen (*P* < .001), and mechanical ventilation (*P* = .003), they more often had a coinciding diagnosis of MIS-C/multisystem inflammatory syndrome in adults (*P* < .001) and required ICU admission at a similar frequency to adults (Table 1).

**Table 1.** Baseline Characteristics, Clinical Features, and Outcomes of COVID-19 in Hospitalized Children vs Adults

Variables^a^	N	Total	Children (0–21 y)	Adults (>21 y)	*P* Value
Total, n	…	**1560**	**885**	**675**	
Age, y, median (IQR)	…	17 (21.8–69.5)	5.8 (0.7–14.2)	62 (48–74]	**<**.**001**
Sex, female	1560	716 (45.9)	399 (45.1)	317 (47.0)	.460
Race	1489	…	…	…	**<**.**001**
Asian	…	142 (9.5)	52 (6.2)	90 (14.0)	
Black or African American	…	389 (26.1)	156 (18.5)	233 (36.2)	
White	…	485 (32.6)	266 (31.5)	219 (34.0)	
Other	…	473 (31.8)	371 (43.9)	102 (15.8)	
Latinx	1480	532 (36.0)	440 (52.9)	92 (14.2)	**<**.**001**
Vaccinated (any COVID-19 vaccine)	1294	319 (24.7)	90 (14.3)	229 (34.5)	**<**.**001**
Comorbidities	**…**	…	…	…	
Autoimmune disease	1556	118 (7.6)	36 (4.1)	82 (12.2)	**<**.**001**
Active malignancy	1555	170 (10.9)	70 (7.9)	100 (14.9)	**<**.**001**
Immunocompromised (HIV/AIDS, transplant, etc.)	1556	324 (20.8)	143 (16.2)	181 (26.9)	**<**.**001**
Hypertension	1555	440 (28.3)	40 (4.5)	400 (59.6)	**<**.**001**
Obesity	1541	417 (27.1)	143 (16.3)	274 (41.5)	**<**.**001**
Diabetes	1554	238 (15.3)	31 (3.5)	207 (30.9)	**<**.**001**
Cardiac disease	1556	268 (17.2)	86 (9.7)	182 (27.1)	**<**.**001**
Lung disease (asthma, OSA, COPD, etc.)	1558	398 (25.5)	216 (24.4)	182 (27.0)	.237
Comorbidities (any)	1560	1087 (69.7)	472 (53.3)	615(91.1)	**<**.**001**
Omicron infection	1544	610 (39.5)	438 (49.5)	172 (26.1)	**<**.**001**
Co-infections	1138	314 (27.6)	201 (24.9)	113 (34.4)	.**001**
Site	1560	…	…	…	**<**.**001**
San Francisco	…	449 (28.8)	45 (5.1)	404 (59.9)	
Atlanta	…	425 (27.2)	156 (17.6)	269 (39.9)	
Philadelphia	…	13 (0.8)	13 (1.5)	0	
Houston	…	193 (12.4)	193 (21.8)	0	
Los Angeles	…	480 (30.8)	478 (54.0)	2 (0.3)	
Symptoms	**…**	…	…	…	
Symptomatic for COVID-19	1558	1547 (99.3)	875 (98.9)	672 (99.9)	.**022**
Fever	1215	1022 (84.1)	689 (90.1)	333 (74.0)	**<**.**001**
Chills	685	193 (28.2)	47 (12.7)	146 (46.4)	**<**.**001**
Headache	690	208 (30.1)	86 (22.1)	122 (40.7)	**<**.**001**
Fatigue	801	470 (58.7)	207 (47.6)	263 (71.9)	**<**.**001**
Cough	1105	876 (79.3)	469 (75.0)	407 (84.8)	**<**.**001**
Shortness of Breath/dyspnea	1029	759 (73.8)	339 (63.5)	420 (84.9)	**<**.**001**
Nasal Congestion/rhinorrhea	849	429 (50.5)	362 (64.3)	67 (23.4)	**<**.**001**
Sore throat	665	129 (19.4)	69 (18.0)	60 (21.3)	.293
Myalgias	691	225 (32.6)	66 (17.7)	159 (50.2)	**<**.**001**
Nausea/vomiting	806	408 (50.6)	266 (54.2)	142 (45.1)	.**012**
Diarrhea	738	288 (39.0)	136 (32.9)	152 (46.8)	**<**.**001**
Conjunctivitis	601	22 (3.7)	21 (5.8)	1 (0.4)	.**001**
Anosmia/dysgeusia	620	73 (11.8)	13 (3.7)	60 (22.5)	**<**.**001**
Outcomes	**…**	…	…	…	
Duration of hospitalization, days, median (IQR)	1521	4 (2–9)	3 (1–6)	7 (4–16)	**<**.**001**
Supplemental oxygen (any)	1560	940 (61.0)	411 (46.5)	529 (80.6)	**<**.**001**
High-flow supplemental oxygen	1366	474 (34.7)	229 (27.3)	245 (46.5)	**<**.**001**
Mechanical ventilation	1533	251 (16.4)	123 (14.0)	128 (19.6)	.**003**
ICU admission	1534	432 (28.2)	233 (26.4)	199 (30.6)	.067
MIS-C/MIS-A	1525	24 (1.6)	22 (2.5)	2 (0.3)	.**001**
Death	1557	81 (5.2)	5 (0.6)	76 (11.3)	**<**.**001**

Abbreviations: AIDS, acquired immunodeficiency syndrome; COPD, chronic obstructive pulmonary disease; HIV, human immunodeficiency virus; ICU, intensive care unit; IQR, interquartile range; MIS-A, multisystem inflammatory syndrome in adults; MIS-C, multisystem inflammatory syndrome in children; OSA, obstructive sleep apnea.

^a^All values represent number of patients (%) unless otherwise indicated. The denominator for percentages was the number of patients for whom data were available (eg, omitted missing data).

After adjusting for sex, race, ethnicity, comorbidities, Omicron status, vaccination status, co-infections, and study site, children had significantly increased odds of receiving high-flow oxygen (adjusted odds ratio [aOR], 2.18; 95% confidence interval [CI], 1.29–3.67), but decreased odds of receiving (any) supplemental oxygen (aOR, 0.57; 95% CI, .35–.92) and death (aOR, 0.01; 95% CI, <.001–.58) (Table 2).

**Table 2.** Unadjusted and Adjusted^a^ Odds Ratios of Severe Outcomes of Children vs Adults

Odds of Severe Outcome in Children Versus Adults (reference), OR (95%)		
…	Unadjusted Odds Ratio	Adjusted Odds Ratio
Supplemental oxygen (any)	0.21 (0.17, 0.27)	**0.57 (0.35, 0.92)**
High-flow supplemental oxygen	0.43 (0.34, 0.54)	**2.18 (1.29, 3.67)**
Mechanical ventilation	0.67 (0.51, 0.87)	0.53 (0.26, 1.12)
ICU admission	0.81 (0.65, 1.02)	1.40 (0.84, 2.18)
Death	0.05 (0.02, 0.11)	**0.01 (<0.01, 0.58)**

Abbreviations: ICU, intensive care unit; OR, odds ratio.

^a^Multivariable analysis adjusted for sex, race, ethnicity, vaccination, co-infection, Omicron Status, and study site.

### Comparisons of Pediatric Age Strata

Of the 885 children in the analysis population, 238 were aged <1 year, 185 were aged 1–4 years, 162 were aged 5–11 years, 224 were aged 12–17 years, and 76 were aged 18–21 years. Younger children were less commonly vaccinated (0.6%, 2.1%, 18.1%, 24.5%, 49.2% with increasing age strata) and more commonly infected with the Omicron variant (60.5%, 59.5%, 48.8%, 33.5%, 39.5% with increasing age strata). Children aged <1 year and 1–4 years had fewer comorbidities compared to older children (*P* < .05 for most comparisons), except for cardiac disease. Across the age groups, frequencies of supplemental oxygen (31.5%, 40.5%, 46.6%, 64.7%, and 56.6%; overall *P* < .001), high-flow oxygen (18.7%, 25.0%, 26.0%, 38.1%, and 30.0%; overall *P* < .001), mechanical ventilation (8.1%, 10.3%, 15.4%, 21.6%, and 15.8%; overall *P* < .001) requirement and ICU admission (20.6%, 26.0%, 24.1%, 35.4%, 23.7%; overall *P* = .007) were highest among children 12–17 years of age. Children aged 5–11 years and 12–17 years were also most frequently diagnosed with MIS-C (6.3% and 3.2%, respectively) (Table 3). Infants aged <1 year most commonly had co-infections, the majority of which were either respiratory viral (36.2%) or urinary tract infections (39.7%) (Supplementary Table 4).

**Table 3.** Baseline Characteristics, Clinical Features, and Outcomes of COVID-19 in Pediatric age Strata

Variables^a^	<1 y (a)	1–4 y (b)	5–11 y (c)	12–17 y (d)	18–21 y (e)	*P* Value Overall	*P* Value (d) Versus (a)	*P* Value (d) Versus (b)	*P* Value (d) Versus (c)	*P* Value (d) Versus (e)
Total, n	238	185	162	224	76	…	…	…	…	…
Age, y, median (IQR)	0.1 (0.0–0.4)	2.1 (1.3, 3.0)	8.5 (6.4–10.0)	15.0 (13.7–16.5)	19.4 (18.5–20.8)	**…**	**…**	**…**	**…**	**…**
Sex, Female	109 (45.8)	73 (39.5)	71 (43.8)	109 (48.7)	37 (48.7)	…	…	…	…	…
Race	…	…	…	…	…	.**001**	.**001**	.256	.774	.**035**
Asian	11 (4.9)	16 (9.0)	11 (7.3)	11 (5.1)	3 (4.0)	…	…	…	…	…
Black or African American	26 (11.5)	32 (18.0)	33 (21.9)	51 (23.8)	14 (18.7)	…	…	…	…	…
White	64 (28.2)	58 (32.6)	55 (36.4)	73 (34.1)	16 (21.3)	…	…	…	…	…
Other	126 (55.5)	72 (40.5)	52 (34.4)	79 (36.9)	42 (56.0)	…	…	…	…	…
Latinx	139 (62.9)	88 (50.0)	62 (41.3)	104 (49.5)	47 (62.7)	**<**.**001**	.**005**	.926	.124	.**050**
Vaccinated (Y/N)	1 (0.6)	3 (2.1)	19 (18.1)	36 (24.5)	31 (49.2)	**<**.**001**	**<**.**001**	**<**.**001**	.**230**	**<**.**001**
Comorbidities	**…**	…	…	…	…	…	…	…	…	…
Autoimmune disease	1 (0.4)	7 (3.8)	12 (7.5)	10 (4.5)	6 (7.9)	.**003**	.**004**	.732	.213	.250
Active malignancy	0 (0.0)	13 (7.0)	22 (13.7)	21 (9.4)	14 (18.4)	**<**.**001**	**<**.**001**	.392	.187	.**034**
Immunocompromised (AIDS, transplant, multiple myeloma, etc.)	6 (2.5)	29 (15.7)	36 (22.4)	45 (20.1)	27 (35.5)	**<**.**001**	**<**.**001**	.249	.589	.**007**
Hypertension	1 (0.4)	8 (4.3)	9 (5.6)	12 (5.4)	10 (13.2)	**<**.**001**	.**001**	.629	.921	.**024**
Obesity	0 (0.0)	5 (2.7)	21 (13.1)	90 (40.4)	27 (35.5)	**<**.**001**	.**001**	.**001**	.**001**	.460
Diabetes	0 (0.0)	0 (0.0)	1 (0.6)	20 (8.9)	10 (13.2)	**<**.**001**	.**001**	.**001**	.**001**	.288
Cardiac disease	18 (7.6)	25 (13.5)	14 (8.7)	24 (10.7)	5 (6.6)	.232	.239	.386	.514	.292
Lung Disease (asthma, OSA, COPD, etc.)	9 (3.8)	49 (26.5)	57 (35.2)	74 (33.0)	27 (35.5)	**<**.**001**	.**001**	.15	.659	.691
Omicron infection	144 (60.5)	110 (59.5)	79 (48.8)	75 (33.5)	30 (39.5)	**<**.**001**	.**001**	.**001**	.**003**	.344
Symptoms	**…**	…	…	…	…	…	…	…	…	…
Fever	191 (92.7)	154 (91.7)	121 (86.4)	174 (88.3)	49 (90.7)	.301	.131	.292	.604	.617
Chills	3 (4.4)	2 (2.6)	8 (11.0)	26 (20.6)	8 (29.6)	**<**.**001**	.**003**	**<**.**001**	.081	.307
Headache	0 (0.0)	5 (6.3)	29 (34.1)	45 (32.6)	7 (31.8)	**<**.**001**	**<**.**001**	**<**.**001**	.816	.941
Fatigue	33 (40.7)	38 (40.0)	44 (51.2)	74 (52.1)	18 (58.1)	.153	.102	.067	.889	.547
Cough	114 (75.5)	90 (69.8)	84 (76.4)	135 (75.0)	46 (83.6)	.379	.917	.308	.793	.183
Shortness of Breath/dyspnea	70 (58.3)	70 (60.3)	52 (55.3)	116 (72.1)	31 (72.1)	.**026**	.**016**	.**041**	.**007**	.995
Nasal Congestion/rhinorrhea	128 (80.0)	82 (69.5)	52 (52.0)	69 (48.6)	31 (72.1)	**<**.**001**	**<**.**001**	**<**.**001**	.602	.**007**
Sore throat	0 (0.0)	5 (6.3)	21 (26.3)	33 (25.4)	10 (35.7)	**<**.**001**	**<**.**001**	**<**.**001**	.889	.265
Myalgias	0 (0.0)	2 (2.6)	15 (20.3)	40 (30.8)	9 (33.3)	**<**.**001**	**<**.**001**	**<**.**001**	.104	.794
Nausea/vomiting	45 (48.4)	63 (57.8)	59 (55.7)	83 (56.1)	16 (45.7)	.533	.244	.784	.947	.268
Diarrhea	33 (38.8)	22 (25.6)	21 (25.3)	52 (39.1)	8 (30.8)	.093	.968	.**039**	.**037**	.423
Conjunctivitis	5 (7.3)	2 (2.6)	10 (13.0)	3 (2.6)	1 (4.6)	.**025**	.132	.985	.**005**	.616
Anosmia or Dysgeusia	0 (0.0)	1 (1.3)	3 (4.2)	7 (5.9)	2 (9.1)	.124	.**044**	.118	.597	.579
Outcomes	**…**	…	…	…	…	…	…	…	…	…
Duration of hospitalization, days Median (IQR)	2 (1–3)	2 (1–5)	3 (2–6)	4 (2–7)	4 (2–7)	**<**.**001**	**<**.**001**	**<**.**001**	**<**.**001**	**<**.**001**
supplemental oxygen (any)	75 (31.5)	75 (40.5)	75 (46.6)	145 (64.7)	43 (56.6)	**<**.**001**	**<**.**001**	**<**.**001**	**<**.**001**	.204
High-flow supplemental oxygen	42 (18.7)	44 (25)	39 (26.0)	83 (38.1)	21 (30.0)	**<**.**001**	**<**.**001**	.**006**	.**016**	.221
Mechanical ventilation	19 (8.1)	19 (10.3)	25 (15.4)	48 (21.6)	12 (15.8)	**<**.**001**	**<**.**001**	.**002**	.127	.274
ICU admission	49 (20.6)	48 (26.0)	39 (24.1)	79 (35.4)	18 (23.7)	.**007**	**<**.**001**	.**040**	.**020**	.059
MIS-C/MIS-A	1 (0.4)	4 (2.2)	10 (6.3)	7 (3.2)	0 (0.0)	.**003**	**…**	**…**	**…**	**…**
Death	1 (0.4)	1 (0.5)	0 (0.0)	2 (0.9)	1 (1.3)	.701	.529	.678	.229	.748

Abbreviations: AIDS, acquired immunodeficiency syndrome; COPD, chronic obstructive pulmonary disease; HIV, human immunodeficiency virus; ICU, intensive care unit; IQR, interquartile range; MIS-A, multisystem inflammatory syndrome in adults; MIS-C, multisystem inflammatory syndrome in children; OSA, obstructive sleep apnea.

Overall *P* values were calculated using 1-way analysis of variance. Paired comparisons were made using chi-square tests comparing each group to the reference. Group 1, < 1 year; group 2, 1–4 years; group 3, 5–11 years; group 4, 12–17 years (reference); group 5, 18–21 years of age.

^a^All values represent number of patients (%) unless otherwise indicated. The denominator for percentages was the number of patients for whom data was available (eg, omitted missing data).

**Figure 1.**
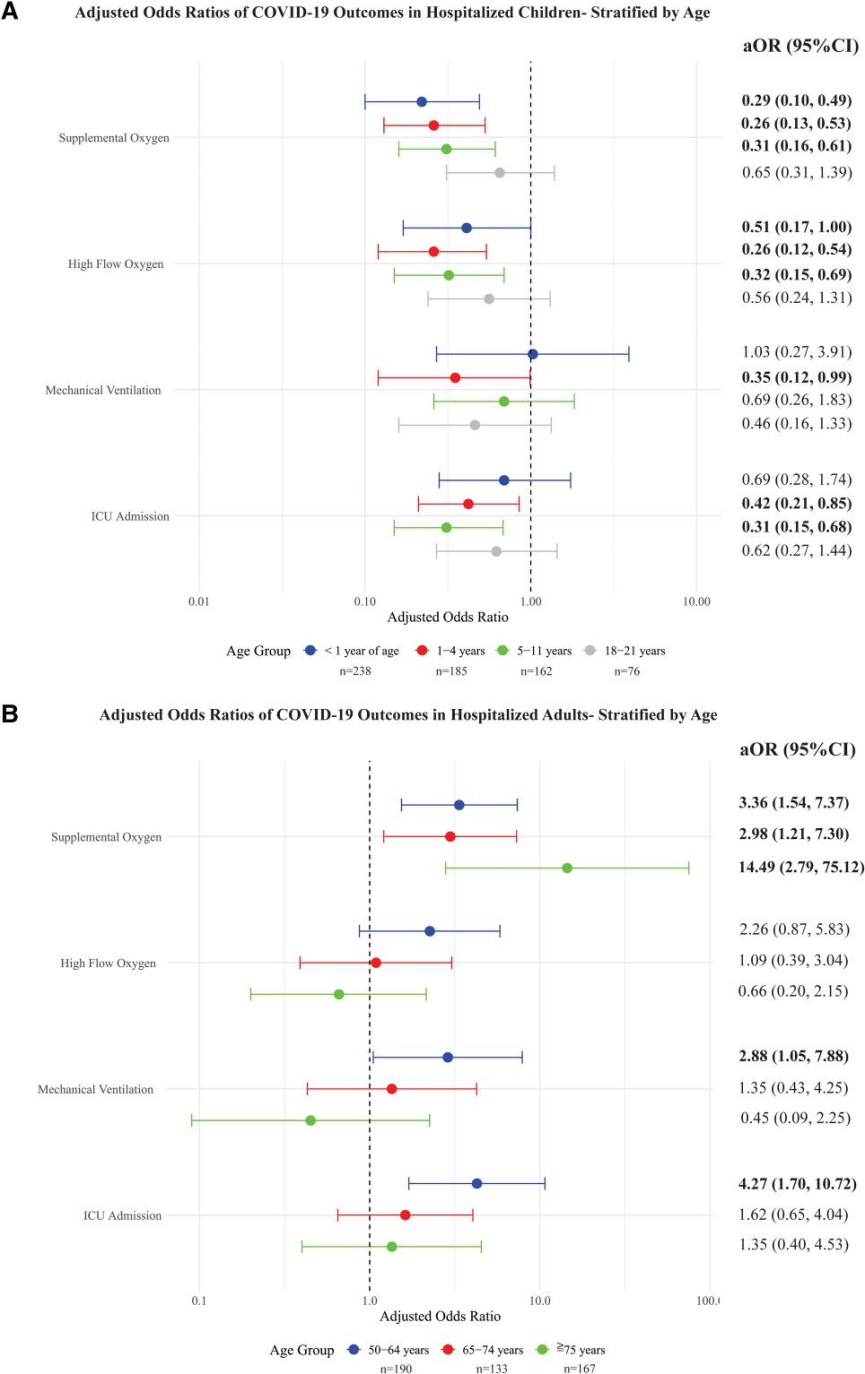
Adjusted odds ratios (aOR) with 95% confidence intervals (CI) of severe outcomes* among (*A*) children and (*B*) adults hospitalized for COVID-19. Multivariate logistic regression analysis was performed to determine odds ratios adjusting for age, sex, race, Latinx ethnicity, vaccination status (yes/no), SARS-CoV-2 variant, co-infection (yes/no), and study site. Adjusted odds ratios are presented for each pediatric age stratum compared to children aged 12–17 y (n = 224, reference), and for each adult age stratum compared to adults aged 22–49 y (n = 185, reference). *The adjusted odds of death was not estimable because of low quantities of this outcome among the age strata. Bolded numbers denote statistical significance.

After adjusting for confounding variables, children aged <1 years, 1–4 years, and 5–11 years had significantly decreased odds of requiring supplemental oxygen (<1 year: aOR, 0.22; 95% CI, .10–.49; 1–4 years: aOR, 0.26; 95% CI, .13–.53; 5–11 years: aOR, 0.31; 95% CI, .16–.61) and receiving high-flow oxygen (<1 year: aOR, 0.41; 95% CI, .17–1.00; 1–4 years: aOR, 0.26; 95% CI, .12–.54; 5–11 years: aOR, 0.32; 95% CI, .15–.69) compared to children 12–17 years (reference group) (Table 4, Figure 1). Children aged 1–4 years and 5–11 years also had decreased odds of requiring ICU admission (1–4 years: aOR, 0.42; 95% CI, .21–.85; 5–11 years: aOR, 0.31; 95% CI, .15–.68) (Table 4, Figure 1). Overall, adolescents aged 12–17 years experienced the greatest disease severity among the pediatric age strata (Figure 1).

**Table 4.** Adjusted Odds Ratios (With 95% CIs) of Severe Outcomes Between age Strata

Age Strata	Supplemental Oxygen (any)	High-Flow Supplemental Oxygen	Mechanical Ventilation	ICU Admission
Children Adjusted Odds Ratio For Severe Outcomes (95% CI)^a^				
<1 y	**0.22 (0.10, 0.49)**	**0.41 (0.17, 1.00)**	1.03 (0.27, 3.91)	0.69 (0.28, 1.74)
1–4 y	**0.26 (0.13, 0.53)**	**0.26 (0.12, 0.54)**	**0.35 (0.12, 0.99)**	**0.42 (0.21, 0.85)**
5–11 y	**0.31 (0.16, 0.61)**	**0.32 (0.15, 0.69)**	0.69 (0.26, 1.83)	**0.31 (0.15, 0.68)**
12–17 y (reference)	1	1	1	1
18–21 y	0.65 (0.31, 1.39)	0.56 (0.24, 1.31)	0.46 (0.16, 1.33)	0.62 (0.27, 1.44)
Adults adjusted odds ratios for severe outcomes (95% CI)				
22–49 y (reference)	1	1	1	1
50–64 y	**3.36 (1.54, 7.37)**	2.26 (0.87, 5.83)	**2.88 (1.05, 7.88)**	**4.27 (1.70, 10.72)**
65–74 y	**2.98 (1.21, 7.30)**	1.09 (0.39, 3.04)	1.35 (0.43, 4.25)	1.62 (0.65, 4.04)
≥75 y	**14.49 (2.79, 75.12)**	0.66 (0.20, 2.15)	0.45 (0.09, 2.25)	1.35 (0.40, 4.53)

Abbreviation: CI, confidence interval.

^a^Adjusted odds of death was not estimable because of low quantities of this outcome among the age strata. Bolded numbers denote statistical significance.

### Comparisons of Adult age Strata

Of the 675 adults in the analysis population, 185 were aged 22–49 years, 190 were 50–64 years, 133 were 65–74 years, and 167 were ≥75 years (Table 5). Older adults were more commonly vaccinated (22.7%, 24.6%, 43.5%, 51.8% with increasing age strata) and infected with Omicron (15.9%, 22.7%, 27.7%, 39.8% with increasing strata). Overall, 53.1% of young adults aged 22–49 years were obese, significantly more than adults aged 65–74 years (*P* = .003) and ≥75 (*P* ≤ .001) years. Compared to adults aged 22–49 years, older age groups had significantly more hypertension, diabetes, and cardiac disease (*P* < .001 for all comparisons). Older adults received supplemental oxygen at a higher frequency than younger adults aged 22–49 years (*P* < .01 for all comparisons). Across the groups, adults aged 50–64 years and 65–74 years more commonly required ICU admission (*P* = .007) and experienced more death (*P* = .040) (Table 5).

**Table 5.** Baseline Characteristics, Clinical Symptoms, and Outcomes of COVID-19 in Adult age Strata

Variables^a^	22–49 Y (a)	50–64 Y (b)	65–74 Y (c)	≥75 Y (d)	*P* Value (overall)	*P* Value (a) Versus (b)	*P* Value (a) Versus (c)	*P* Value (a) Versus (d)
Total, no.	185	190	133	167	…	…	…	…
Age, median (IQR)	37 (29–43)	57 (53–61)	69 (66–71)	82 (78–89)	**…**	**…**	**…**	**…**
Sex, female (%)	99 (53.5)	79 (41.6)	56 (42.1)	83 (49.7)	…	…	…	…
Race	…	…	…	…	**<**.**001**	.**043**	.**020**	**<**.**001**
Asian	20 (11.6)	17 (9.4)	16 (12.4)	37 (22.8)	…	…	…	…
Black or African American	76 (44.2)	83 (45.9)	45 (34.9)	29 (17.9)	…	…	…	…
White	36 (20.9)	56 (30.9)	47 (36.4)	80 (49.4)	…	…	…	…
Other	40 (23.3)	25 (13.8)	21 (16.3)	16 (9.9)	…	…	…	…
Latinx	32 (18.4)	28 (15.3)	22 (17.1)	10 (6.2)	.**001**	.435	.764	**<**.**001**
Vaccinated (Y/N)	41 (22.7)	46 (24.6)	57 (43.5)	85 (51.8)	**<**.**001**	.660	**<**.**001**	**<**.**001**
Comorbidities	…	…	…	…	…	…	…	…
Autoimmune disease	20 (10.9)	19 (10.0)	17 (12.9)	26 (15.6)	.398	.769	.596	.199
Active malignancy	17 (9.3)	22 (11.6)	25 (18.9)	36 (21.6)	.**004**	.480	.**014**	.**002**
Immunocompromised (AIDS, transplant, multiple myeloma, etc.)	49 (26.8)	61 (32.1)	36 (27.3)	35 (21.0)	.132	.259	.922	.203
Hypertension	48 (26.5)	120 (63.2)	95 (71.4)	137 (82.0)	**<**.**001**	**<**.**001**	**<**.**001**	**<**.**001**
Obesity	95 (53.1)	83 (44.4)	47 (35.9)	49 (29.9)	**<**.**001**	.097	.**003**	**<**.**001**
Diabetes	30 (16.5)	69 (36.5)	51 (38.6)	57 (34.1)	**<**.**001**	**<**.**001**	**<**.**001**	**<**.**001**
Cardiac disease	15 (8.2)	50 (26.5)	42 (31.6)	75 (44.9)	**<**.**001**	**<**.**001**	**<**.**001**	**<**.**001**
Lung disease (asthma, OSA, COPD, etc.)	37 (20.2)	54 (28.4)	42 (31.6)	49 (29.3)	.096	.065	.**021**	.**048**
Omicron infection	29 (15.9)	41 (22.7)	36 (27.7)	66 (39.8)	**<**.**001**	.105	.**012**	**<**.**001**
Symptoms	…	…	…	…	…	…	…	…
Fever	109 (81.3)	105 (70.0)	61 (75.3)	58 (68.2)	.088	.027	.292	.**026**
Chills	56 (58.3)	48 (43.6)	23 (39.0)	19 (38.0)	.**036**	.**035**	.**019**	.**020**
Headache	43 (46.2)	44 (40.7)	25 (41.7)	10 (25.6)	.182	.433	.579	.**028**
Fatigue	69 (69.0)	79 (65.3)	51 (75.0)	64 (83.1)	.**043**	.559	.398	.**031**
Cough	101 (80.8)	131 (87.3)	77 (81.9)	98 (88.3)	.267	.138	.834	.114
Shortness of Breath/dyspnea	114 (83.2)	128 (85.9)	81 (81.8)	97 (88.2)	.557	.528	.780	.271
Nasal congestion/rhinorrhea	23 (26.1)	14 (14.1)	17 (31.5)	13 (28.9)	.**050**	.**040**	.**492**	.**735**
Sore throat	24 (27.6)	12 (12.2)	12 (21.8)	12 (28.6)	.**042**	.**009**	.442	.907
Myalgias	66 (61.7)	47 (45.6)	29 (46.0)	17 (38.6)	.**026**	.**020**	.**047**	.**010**
Nausea/vomiting	50 (52.6)	52 (44.8)	26 (44.8)	14 (30.4)	.103	.259	.349	.013
Diarrhea	43 (44.3)	56 (48.7)	24 (43.6)	29 (50.0)	.834	.526	.934	.493
Conjunctivitis	0 (0.0)	1 (1.1)	0 (0.0)	0 (0.0)	.065	.376	—	—
Anosmia/dysgeusia	22 (27.2)	25 (25.0)	7 (14.0)	6 (16.7)	.246	.742	.078	.220
Outcomes	…	…	…	…	…	…	…	…
Duration of hospitalization, days, median (IQR)	5 (3–11)	7(4–15)	9.5 (6–20)	10 (5–18)	**<**.**001**	.**018**	**<**.**001**	**<**.**001**
Supplemental oxygen (any)	122 (67.8)	153 (83.2)	110 (85.9)	144 (87.8)	**<**.**001**	.**001**	**<**.**001**	**<**.**001**
High-flow supplemental oxygen	52 (42.6)	83 (54.6)	52 (47.3)	58 (40.6)	.078	.**049**	.477	.734
Mechanical ventilation	36 (20.2)	38 (20.9)	28 (21.7)	26 (16.0)	.579	.878	.753	.307
ICU admission	46 (26.3)	70 (38.3)	45 (35.2)	38 (23.2)	.**007**	.**016**	.**096**	.**507**
Death	13 (7.0)	20 (10.6)	23 (17.3)	20 (12.0)	.**040**	.226	.**004**	.112

Abbreviations: AIDS, acquired immunodeficiency syndrome; COPD, chronic obstructive pulmonary disease; HIV, human immunodeficiency virus; ICU, intensive care unit; IQR, interquartile range; MIS-A, multisystem inflammatory syndrome in adults; MIS-C, multisystem inflammatory syndrome in children; OSA, obstructive sleep apnea.

^a^All values represent number of patients (%) unless otherwise indicated. The denominator for percentages was the number of patients for whom data was available (eg, omitted missing data). Overall *P* values were calculated using 1-way analysis of variance. Paired comparisons were made using chi-square tests comparing each group to the reference. Group 1, 22–49 years (reference); group 2, 50–64 years; group 3, 65–74 years; group 4, ≥ 75 years of age.

After adjusting for confounding variables, adults aged 50–64 years, 65–74 years, and ≥75 years had higher odds of receiving supplemental oxygen compared to adults aged 22–49 years (reference group) (aOR, 3.36; 95% CI, 1.54–7.37; aOR, 2.98; 95% CI, 1.21–7.30; aOR, 14.49; 95% CI, 2.79–75.12) (Table 4, Figure 1). Adults aged 50–64 years also had higher odds of requiring mechanical ventilation (aOR, 2.88; 95% CI, 1.05–7.88) and ICU admission (aOR, 4.27; 95% CI, 1.7–10.72) compared to adults aged 22–49 years (Table 4).

## DISCUSSION

In this multicenter cohort analysis of children and adults hospitalized for COVID-19, we compared risk factors, symptoms, and outcomes between children and adults and across age strata. After adjusting for confounding variables, we found that children had decreased odds of requiring supplemental oxygen overall (aOR, 0.57; 95% CI, .35–.92) and decreased odds of death (aOR, 0.01; 95% CI, <.01–.58) compared to adults. These results support observations in the literature that children overall experience less severe COVID-19 disease compared to adults. Interestingly, children in our study had increased adjusted odds of receiving high-flow oxygen (aOR, 2.18; 95% CI, 1.29–3.67) compared to adults, which may be because this modality is more commonly used to support ventilation in the pediatric population.

Among the pediatric age strata, adolescents aged 12–17 years experienced the most severe disease and most commonly had underlying comorbidities. Severe outcomes were observed at statistically similar rates in the oldest adolescents, aged 18–21 years. Compared to adolescents aged 12–17 years, children 1–4 years and 5–11 years had significantly decreased odds of supplemental oxygen, high-flow supplemental oxygen, and ICU admission. Interestingly, although infants aged <1 year also had decreased odds of supplemental oxygen requirement, they had similar odds of mechanical ventilation and ICU admission. Thus, our results showed the highest odds of severe outcomes in infants and adolescents, as has been demonstrated in other small studies [6, 14].

Among the adult age strata, adults 50–64 years, 65–74 years, and ≥75 years had increased odds of requiring supplemental oxygen compared to younger adults. Interestingly, adults aged 50–64 years had the highest odds of requiring mechanical ventilation and ICU admission after adjusting for confounding variables. These results differ from previous reports that, across all groups, adults ≥65 years of age experience the highest proportion of ICU admission, mechanical ventilation, and death [4]. This may be explained by a hospitalization bias for adults in this age stratum, or a decreased likelihood that older adults would undergo intubation and mechanical ventilation because of frailty or goals of care. Another explanation for our findings is that we did not capture deaths after discharge or hospice deaths, which may have been higher among the oldest adults with significant comorbidities.

Compared to adults, children more commonly experienced fever and upper respiratory symptoms, but less commonly experienced shortness of breath and other systemic symptoms. This corroborates previous studies suggesting that children may experience milder and distinct clinical features compared to adults [15–17]. In our study, we also observed that infants had the highest frequency of nasal congestion/rhinorrhea and conjunctivitis, whereas older children had a higher proportion of chills, headache, shortness of breath/dyspnea, and myalgias. Differences in the clinical features observed in our study could be explained in part by co-infections. The most common co-infections observed in our study were other respiratory viruses, and these may have modified the clinical presentation. A previous multicenter analysis reported that severe COVID-19 outcomes occurred in 45.6% of cases with co-infection compared to 22.1% without co-infection [18]. Further research is needed to understand how co-infections with other respiratory viruses increase COVID-19 severity and the diversity of symptom manifestations in children.

Regarding risk factors for severe COVID-19 outcomes, children had fewer comorbidities than adults, except for lung disease. A systematic review from the US Centers for Disease Control and Prevention indicated that underlying asthma is associated with hospitalization and increased ICU admission among COVID-19 children and adults [19]. Although younger children are less likely to have certain comorbidities, our analysis revealed that older children, especially those who are immunocompromised, obese, and/or with lung disease, are at risk for severe COVID-19 outcomes. Among the adult strata, obesity was a risk factor for severe COVID-19 in younger adults, whereas active malignancy, hypertension, and cardiac disease were risk factors in older adults, consistent with prior reports [20, 21].

Our study adds to the literature by directly comparing clinical manifestations and outcomes of COVID-19 between children and adults across multiple age strata. Current studies comparing children and adults are often limited by small sample size or single-site analyses [22, 23]. Our larger sample size and multicenter design makes our results potentially more generalizable to communities across the United States. However, there are several limitations to our study. First, this was a secondary analysis of patients included at 5 participating PreVAIL sites from March 2020 to May 2023. Patients in the database do not include all patients who tested positive for SARS-CoV-2 at their respective hospitals. Second, data collection was limited to manual chart abstraction, and thus many patients had missing data elements, which we attempted to address with sensitivity analyses. Third, although we adjusted for co-infections in our analysis of patient outcomes, our data did not differentiate preexisting co-infections from secondary co-infections. Fourth, there may have been hospitalization bias for children and adults because patients with underlying comorbidities and/or at the extremes of age (infants and elderly adults) are more likely to be hospitalized with milder disease. For example, young infants in our study may have been admitted based on febrile infant protocols and not for COVID-19 severity alone. Fifth, reporting bias may have confounded results because younger children are inherently less likely to report certain comorbidities and symptoms than adults. Finally, we did not measure prematurity nor all other comorbidities that may be associated with severe COVID-19 [20]. More investigation into the effects of comorbidities among different age strata are important areas for future research.

## CONCLUSION

In conclusion, adults hospitalized for COVID-19 had more severe outcomes overall. Adolescents experienced the greatest disease severity compared to younger children. Children and adults hospitalized with COVID-19 differed in risk factors and symptom presentation across different age strata. Understanding differential risk factors and disease manifestations can inform targeted interventions to prevent severe COVID-19 in children and adults.

## Supplementary Material

ofae443_Supplementary_Data

## Supplementary Data

Supplementary materials are available at *Open Forum Infectious Diseases* online. Consisting of data provided by the authors to benefit the reader, the posted materials are not copyedited and are the sole responsibility of the authors, so questions or comments should be addressed to the corresponding author.

## Notes

***Acknowledgments.*** The authors thank the research coordinators and hospital and laboratory staff at all the sites who made this study possible.

***Funding*.** This study was funded by the Eunice Kennedy Shriver National Institute of Child Health and Human Development (NICHD) grant R61HD105618 (to J. D. B., R. A. L., A. R. O. J., P. J. P., S. D., A. V. A., R. A. L., C. Y. C., and C. A. R.), Centers for Disease Control and Prevention contract award BAA 75D301–22-R-72097 (to C. Y. C.), and Centers for Disease Control and Prevention contract award BAA75D301-21-R-71738 (to P. J. P.). The funders had no role in study design, data collection and analysis, decision to publish, or preparation of the manuscript.

***Author Contributions*.** Conception and design: A. C., J. D. B., P. J. P., S. D., A. V. A., R. A. L., C. Y. C., C. A. R. Acquisition of data: N. B., C. C., H. H., M. A. P., M. O., A. F., S. B., V. S., A. S. G., M. G., D. H., C. K., V. K., Y. F. W., P. Y. C., J. F. V. Analysis and interpretation of data: G. L., K. G., N. B., A. T., C. Y. C., C. A. R. Wrote the manuscript: G. L., K. G., C. Y. C., C. A. R. All authors reviewed and revised the manuscript, approved the final version, and agree to be accountable for all aspects of the study.
