# Supplementary material for: Clinical Features and Outcomes of Pediatric and Adult Patients Hospitalized for Coronavirus Disease 2019: A Comparison Across Age Strata
Source: Open Forum Infect Dis. 2024 Aug 10;11(8):ofae443. doi: 10.1093/ofid/ofae443 (PMC11342389; doi:10.1093/ofid/ofae443)

**Supplementary Material**

**Title: CLINICAL FEATURES AND OUTCOMES OF PEDIATRIC AND ADULT PATIENTS HOSPITALIZED FOR COVID-19: A COMPARISON ACROSS AGE STRATA**

**Authors:** Grace X. Li, Komal Gopchandani, Noah Brazer, Ashley Tippett, Chris Choi, Hui-Mien Hsiao, Miriam Oseguera, Abiodun Foresythe, Sanchita Bhattacharya, Venice Servellita, Alicia Sotomayor Gonzalez, Jennifer K. Spinler, Mark Gonzalez, Dalia Gulick, Colleen Kraft, Vyjayanti Kasinathan, Yun F (Wayne) Wang, Jennifer Dien Bard, Pei Ying Chen, Jessica Flores-Vasquez, Audrey R. Odom John, Paul J. Planet, Sridevi Devaraj, Ananth V. Annapragada, Ruth Ann Luna, Charles Y. Chiu, Christina A. Rostad

**SUPPLEMENTARY METHODS**

Upper respiratory specimens were collected from patients at each participating institution as described below. For this analysis, infections were classified as being Omicron (BA.1 and its sublineages BA.2, BA.2.12.1, BA.3, BA.4, BA.5, XE, BQ.1, BQ.1.1, BF.7., XBB. and XBD) or non-Omicron (all other sequences and indeterminate results).

At Children’s Hospital of Los Angeles (CHLA), residual specimens from the nasopharynx, anterior nares, oropharynx, and saliva swabs that were positive for SARS-CoV-2 by nucleic acid amplification testing (NAAT) were collected and stored at –80°C until reflexed to whole genome sequencing analysis to determine SARS-CoV-2 lineage. Samples were included from inpatients and outpatients, regardless of the reason for testing (CHLA IRB, CHLA-16-00429).

At Children’s Hospital of Philadelphia (CHOP), residual nasopharyngeal swab specimens that were positive for SARS-CoV-2 by NAAT were collected and stored at 4°C, processed within 48 hrs, and stored at -80°C until analysis by whole genome sequencing. For outpatients, swabs were collected based on symptoms; whereas for inpatients and same-day surgery patients, swabs were collected regardless of symptoms (CHOP IRB, 21-018726).

At Emory-affiliated hospitals, including Emory University Hospital, Grady Memorial Hospital, and Children’s Healthcare of Atlanta, residual nasopharyngeal (NP) samples that were positive for SARS-CoV-2 by nucleic acid amplification testing (NAAT) with local Ct values ≤35 were stored at –80°C and were randomly selected and shipped to UCSF for viral genome sequencing. Samples were included from inpatients and outpatients, regardless of the reason for testing (Emory University IRB, STUDY00004820).

At Texas Children’s Hospital (TCH), residual NP samples that tested positive for SARS-CoV-2 by NAAT were obtained from the clinical microbiology laboratory and stored at –80°C until viral genome sequencing. Samples were included from inpatients and outpatients, regardless of the reason for testing (TCH IRB, H-47972).

At University of California San Francisco (UCSF), residual specimens from the nasopharynx, anterior nares, mid-turbinates, or pooled NP and oropharyngeal (OP) swabs that were positive for SARS-CoV-2 by NAAT were collected and stored at –80°C until retrieval from the clinical microbiology laboratory for viral genome sequencing. Samples were included from inpatients and outpatients, regardless of the reason for testing (UCSF IRB, 10-02598).

**SUPPLEMENTARY RESULTS**

**Supplementary Table S1- Sensitivity analysis of clinical symptoms among children and adults hospitalized for COVID-19**

| **Children vs Adults** | **Missing- Excluded** | | | | | | |
| --- | --- | --- | --- | --- | --- | --- | --- |
|  | **Children** | | | **Adults** | | |  |
| **Symptoms** | **Total** | **n** | **%** | **Total** | **n** | **%** | **p-value** |
| Fever | 765 | 689 | 90.1 | 450 | 333 | 74.0 | **<0.001** |
| Chills | 370 | 47 | 12.7 | 315 | 146 | 46.4 | **<0.001** |
| Headache | 390 | 86 | 22.1 | 300 | 122 | 40.7 | **<0.001** |
| Fatigue | 435 | 207 | 47.6 | 366 | 263 | 71.9 | **<0.001** |
| Cough | 625 | 469 | 75.0 | 480 | 407 | 84.8 | **<0.001** |
| Shortness of Breath/Dyspnea | 534 | 339 | 63.5 | 495 | 420 | 84.9 | **<0.001** |
| Nasal Congestion/Rhinorrhea | 563 | 362 | 64.3 | 286 | 67 | 23.4 | **<0.001** |
| Sore Throat | 383 | 69 | 18.0 | 282 | 60 | 21.3 | 0.2933 |
| Myalgias | 374 | 66 | 17.7 | 317 | 159 | 50.2 | **<0.001** |
| Nausea/Vomiting | 491 | 266 | 54.2 | 315 | 142 | 45.1 | **0.012** |
| Diarrhea | 413 | 136 | 32.9 | 325 | 152 | 46.8 | **<0.001** |
| Conjunctivitis | 360 | 21 | 5.8 | 241 | 1 | 0.4 | **<0.001** |
| Anosmia or Dysgeusia | 353 | 13 | 3.7 | 267 | 60 | 22.5 | **<0.001** |
|  | **Missing Set to "No"** | | | | | | |
|  | **Children** | | | **Adults** | | |  |
| **Symptoms** | **Total** | **n** | **%** | **Total** | **n** | **%** | **p-value** |
| Fever | 885 | 689 | 77.9 | 675 | 333 | 49.3 | **<0.001** |
| Chills | 885 | 47 | 5.3 | 675 | 146 | 21.6 | **<0.001** |
| Headache | 885 | 86 | 9.7 | 675 | 122 | 18.1 | **<0.001** |
| Fatigue | 885 | 207 | 23.4 | 675 | 263 | 39.0 | **<0.001** |
| Cough | 885 | 469 | 53.0 | 675 | 407 | 60.3 | **<0.001** |
| Shortness of Breath/Dyspnea | 885 | 339 | 38.3 | 675 | 420 | 62.2 | **<0.001** |
| Nasal Congestion/Rhinorrhea | 885 | 362 | 40.9 | 675 | 67 | 9.9 | **<0.001** |
| Sore Throat | 885 | 69 | 7.8 | 675 | 60 | 8.9 | 0.48 |
| Myalgias | 885 | 66 | 7.5 | 675 | 159 | 23.6 | **<0.001** |
| Nausea/Vomiting | 885 | 266 | 30.1 | 675 | 142 | 21.0 | **<0.001** |
| Diarrhea | 885 | 136 | 15.4 | 675 | 152 | 22.5 | **<0.001** |
| Conjunctivitis | 885 | 21 | 2.4 | 675 | 1 | 0.2 | **<0.001** |
| Anosmia or Dysgeusia | 885 | 13 | 1.5 | 675 | 60 | 8.9 | **<0.001** |

**Supplementary Table S2- Clinical symptoms among infants <3 months of age hospitalized for COVID-19**

| **N=163** | **Missing- Excluded** | | | **Missing Set to "No"** | | |
| --- | --- | --- | --- | --- | --- | --- |
| **Symptoms** | **n** | **Overall** | **%** | **n** | **Overall** | **%** |
| Fever | 141 | 131 | 92.9 | 163 | 131 | 80.4 |
| Chills | 49 | 3 | 6.1 | 163 | 3 | 1.8 |
| Headache | 47 | 0 | 0.0 | 163 | 0 | 0.0 |
| Fatigue | 59 | 26 | 44.1 | 163 | 26 | 16.0 |
| Cough | 92 | 66 | 71.7 | 163 | 66 | 40.5 |
| Shortness of Breath/Dyspnea | 75 | 39 | 52.0 | 163 | 39 | 23.9 |
| Nasal Congestion/Rhinorrhea | 109 | 90 | 82.6 | 163 | 90 | 55.2 |
| Sore Throat | 47 | 0 | 0.0 | 163 | 0 | 0.0 |
| Myalgias | 47 | 0 | 0.0 | 163 | 0 | 0.0 |
| Nausea/Vomiting | 57 | 23 | 40.4 | 163 | 23 | 14.1 |
| Diarrhea | 58 | 22 | 37.9 | 163 | 22 | 13.5 |
| Conjunctivitis | 49 | 4 | 8.2 | 163 | 4 | 2.5 |
| Anosmia or Dysgeusia | 47 | 0 | 0.0 | 163 | 0 | 0.0 |

**Supplementary Table S3- Co-infections in hospitalized children vs adults**

| **Variables, n (%)** | **n** | **Total**  **(n= 1560)** | **Children**  **(n= 885)** | **Adults**  **(n= 675)** | **p-value** |
| --- | --- | --- | --- | --- | --- |
| Co-Infections* | 1138 | 314 (27.6) | 201 (24.9) | 113 (34.4) | **0.001** |
| Type of Co-infection | 277 |  |  |  | **<0.001** |
| Bacteremia/Sepsis |  | 32 (11.6) | 13 (7.3) | 19 (19.0) |  |
| Bacterial pneumonia |  | 16 (5.8) | 1 (0.6) | 15 (15.0) |  |
| Respiratory viral |  | 76 (27.4) | 72 (40.7) | 4 (4.0) |  |
| Gastrointestinal |  | 4 (1.4) | 1 (0.6) | 3 (3.0) |  |
| Skin/soft tissue |  | 5 (1.8) | 3 (1.7) | 2 (2.0) |  |
| Urinary tract infection |  | 60 (21.7) | 42 (23.7) | 18 (18.0) |  |
| Multiple |  | 41(14.8) | 18 (10.2) | 23 (23.0) |  |
| Other respiratory |  | 16 (5.8) | 16 (9.0) | 0 (0.0) |  |
| Other viral |  | 12 (4.3) | 5 (2.8) | 7 (7.0) |  |
| Other |  | 15 (5.4) | 6 (3.4) | 9 (9.0) |  |

* Co-infections were clinical diagnoses abstracted from the electronic medical record. Microbial etiologies were recorded when present. Co-infections that were pre-existing prior to hospitalization were not differentiated from those that were hospital-acquired.

**Supplementary Table S4- Co-infections in hospitalized children across age strata**

| **Variables n (%)** | **< 1 years** | **1-4 years** | **5-11 years** | **12-17 years** | **18-21 years** | **p-value** |
| --- | --- | --- | --- | --- | --- | --- |
| Co-Infections* | 68 (30.4) | 52 (29.7) | 34 (23.3) | 33 (16.8) | 14 (20.6) | **0.009** |
| Type of Co-infections |  |  |  |  |  | **<0.001** |
| Bacteremia/Sepsis | 4 (6.9) | 1 (2.0) | 1 (3.5) | 5 (17.2) | 2 (18.2) |  |
| Bacterial pneumonia | 0 (0.0) | 1 (2.0) | 0 (0.0) | 0 (0.0) | 0 (0.0) |  |
| Respiratory viral | 21 (36.2) | 29 (58.0) | 15 (51.7) | 6 (20.7) | 1 (9.1) |  |
| Gastrointestinal | 1 (1.7) | 0 (0.0) | 0 (0.0) | 0 (0.0) | 0 (0.0) |  |
| Skin/soft tissue | 1 (1.7) | 0 (0.0) | 0 (0.0) | 2 (6.9) | 0 (0.0) |  |
| Urinary tract infection | 23 (39.7) | 6 (12.0) | 3 (10.3) | 6 (20.7) | 4 (36.4) |  |
| Multiple | 6 (10.3) | 3 (6.0) | 3 (10.3) | 5 (17.2) | 1 (9.1) |  |
| Other respiratory | 1 (1.7) | 7 (14.0) | 5 (17.2) | 0 (0.0) | 3 (27.3) |  |
| Other viral | 0 (0.0) | 1 (2.0) | 2 (6.9) | 2 (6.9) | 0 (0.0) |  |
| Other | 1 (1.7) | 2 (4.0) | 0 (0.0) | 3 (10.3) | 0 (0.0) |  |

* Co-infections were clinical diagnoses abstracted from the electronic medical record. Microbial etiologies were recorded when present. Co-infections that were pre-existing prior to hospitalization were not differentiated from those that were hospital-acquired.

**Supplementary Figure S1.** Study Population.*

**
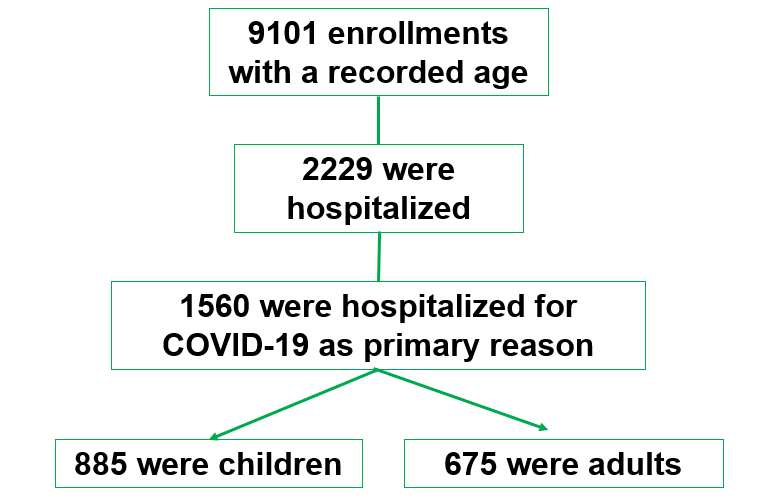
**

* The parent PreVAIL study included children and adults from the 5 participating sites who were diagnosed with SARS-CoV-2 by upper respiratory nucleic acid amplification test (NAAT) and had specimen available for sequencing. “Enrollment” is defined as those included in the PreVAIL study. The present manuscript represents a secondary analysis of those included in the PreVAIL study, and this analysis is limited to those in the database who were hospitalized for COVID-19.

**Supplementary Figure S2.** Distribution of enrollments among children and adults hospitalized for COVID-19 in the analysis population by calendar month.
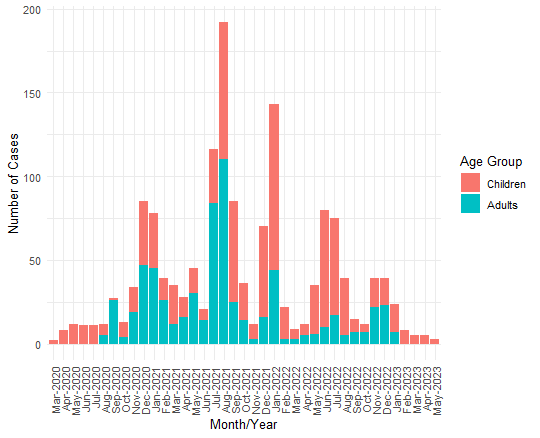

Supplement: ofae443_Supplementary_Data [file ofae443_supplementary_data.docx]
